# Chromosome-level genome assembly of the Asian spongy moths *Lymantria dispar asiatica*

**DOI:** 10.1038/s41597-023-02823-7

**Published:** 2023-12-13

**Authors:** Zhe Xu, Jianyang Bai, Yue Zhang, Lu Li, Mengru Min, Jingyu Cao, Jingxin Cao, Yanchun Xu, Fei Li, Ling Ma

**Affiliations:** 1 College of Wildlife and Protected Area, Northeast Forestry University, Harbin, China; 2 Department of Forest Protection, College of Forestry, Northeast Forestry University, Harbin, China; 3 State Key Laboratory of Rice Biology & Ministry of Agriculture and Rural Affairs Key Laboratory of Molecular Biology of Crop Pathogens and Insects, Institute of Insect Sciences, College of Agriculture and Biotechnology, Zhejiang University, Hangzhou, China

**Keywords:** Genome, Molecular biology, Evolutionary genetics

## Abstract

The Asian spongy moth, *Lymantria dispar asiatica*, is one of the most devastating forestry defoliators. The absence of a high-quality genome limited the understanding of its adaptive evolution. Here, we conducted the first chromosome-level genome assembly of *L. dispar asiatica* using PacBio HIFI long reads, Hi-C sequencing reads and transcriptomic data. The total assembly size is 997.59 Mb, containing 32 chromosomes with a GC content of 38.91% and a scaffold N50 length of 35.42 Mb. The BUSCO assessment indicated a completeness estimate of 99.4% for this assembly. A total of 19,532 protein-coding genes was predicted. Our study provides a valuable genomics resource for studying the mechanisms of adaptive evolution and facilitate an efficient control of *L. dispar asiatica*.

## Background & Summary

The spongy moth, *Lymantria dispar*, is one of the most important forestry pests. It is widely distributed across the temperate forests of the northern hemisphere, such as Europe, China and North America^1,2^. The larvae are destructive polyphagous folivores, and they consume more than 600 plant species ranging from oaks to conifers^3^. They completely defoliate entire trees, resulting in significant ecological and economic losses^4,5^. The spongy moth is divided into Asian (*L. dispar asiatica*) and European (*L. dispar dispar*) species based on origin^6^. Introduced to North America in 1869, the European variant has spread widely over 150 years^7^. The Asia spongy moth poses a greater threat due to its robust reproductive capacity and flight abilities^8^. Females are particularly drawn to lights in ports, often laying their egg masses on cargo and the superstructure of ships^9^. At present, how to effectively control the invasion and spread of the spongy moth has become a global research hotspot^10^.

Chemical control is the primary control method to combat the spongy moth^11^. Since the last century, a variety of insecticides have been used for spongy moth control^12^. Unfortunately, the frequent and extensive use of pesticides not only adversely affects biodiversity but also hastens the development of insecticide resistance. Xenobiotic detoxification is a crucial mechanism enabling insects to resist toxic phytochemicals or pesticides. It depends on the constitutive quantitative changes in the expression and activity of multiple detoxification enzymes, including cytochrome P450s (CYP450s), UDP-glucuronosyltransferase (UGTs), glutathione S-transferases (GSTs) and ATP-binding cassette transporters (ABC) family^13^. Currently, the development of RNAi insecticides targeting these detoxification genes is the focus of the pest control field. However, the lack of genomic information significantly constrains the identification of effective targets in the spongy moth. Additionally, this deficit impedes the understanding of insecticide resistance mechanisms in the spongy moth from the genomic diversity and evolution perspective.

Here, we constructed the first high-quality chromosome-level reference genome of *L. dispar* using PacBio long-read sequencing and Hi-C sequencing technologies. The final genome size was 997.59 Mb with N50 sizes of 35.42 Mb, and 991.35 Mb genome sequences were further clustered and ordered into 32 chromosomes. A total of 19,532 protein-coding genes was predicted in the genome of *L. dispar asiatica*. This chromosome-level genome assembly of *L. dispar asiatica* provides a valuable genomics resource for investigating its evolutionary dynamics and aiding in the control of *L. dispar asiatica*.

## Methods

### Insect rare and sample collection

The egg masses of *L. dispar asiatica* were obtained from a poplar filed in Harbin, Heilongjiang Province and maintained at 4 °C before hatching. Hatched larvae were fed with an artificial diet at 25 ± 1 °C, 14:10 (L:D) photoperiod and 65 ± 5% relative humidity referring to our previous studies^14,15^. The 2nd, 3rd, 4th, 5th instar larvae, pupae, and adult moth were collected separately. The samples were frozen in liquid nitrogen and then stored at −80 °C.

### Genome sequencing and assembly

Genomic DNA was isolated from a fresh female pupa of *Lymantria dispar asiatica* using the sodium dodecyl sulfate (SDS) extraction method^16^. For PacBio long-read sequencing, 8 µg DNA was sheared into fragments of 15–20 kb in length by g-TUBE (Covaris USA) and then purified with AMPure PB Beads. High-fidelity (HiFi) libraries were constructed with SMRTbell Express Template Prep Kit 2.0 and sequenced on Pacbio Sequel IIe platform (Pacifc Biosciences, Menlo Park, USA). A total of 33.02 Gb HiFi reads with N50 sizes of 20,583 bp were obtained using Circular Consensus Sequencing (CCS) mode (Table 1). The PacBio HiFi reads of *L. dispar asiatica* were de novo assembled by using Hifiasm software v0.19.5^17,18^ with default parameters. The draft genome had a total size of 997.53 Mb containing 102 contigs with N50 sizes of 32.048 Mb (Table 2).

**Table 1:** Statistics of the HIFI sequence data used for genome assembly.

sample	Read_base	Read_Number	Read_length(max)	Read_length(mean)	Read_length(N50)
pupa	33,02,05,01,680	16,12,392	49,587	20,479	20,583

**Table 2:** Summary statistics of the *Lymantria* dispar *asiatica* genome assembly.

Contig level	
Assembly length (bp)	99,75,33,159
Longest contig (bp)	4,42,20,918
Number of contigs	102
GC (%)	38.91
Contig N50 (bp)	3,20,48,912
BUSCO	C:99.3%[S:97.8%,D:1.5%],F:0.3%,M:0.4%,n:1367
**Chromosome level**	
Number of chromosomes	32
Chromosome length (bp)	99,75,90,907
Scafold N50 (bp)	3,54,22,674
BUSCO	C:99.4%[S:97.9%,D:1.5%],F:0.2%,M:0.4%,n:1367

### Hi-C scaffolding

To construct Hi-C libraries, the 5th instar female larva of *L. dispar asiatica* was used as inputs following previously described standard protocols^19^. In detail, the larva was cut into small pieces and pulverized in liquid nitrogen. The tissues were cross-linked by 4% formaldehyde solution for 30 mins. After quenching the crosslinking reaction with 2.5 M glycine, tissue sample was centrifuged at 2500 rpm at 4°C for 10 mins. The pellet was washed with 500 μl PBS and then centrifuged for 5 min (2500 rpm). Subsequently, the pellet was resuspended in 20 ul of lysis buffer, followed by twice washing with 100 μl ice cold 1x NEB buffer. The nuclei were collected by centrifuging at 5000 rpm for 5 min, resuspended with 100 μl NEB buffer, and solubilized with dilute SDS. After quenching the SDS with Triton X-100, the samples were digested overnight at 37 °C with a 4-cutter restriction enzyme MboI (400 units). The linked DNA was labelled with biotin-14-dCTP and then ligated by T4 DNA polymerase. The ligated DNA was sheared fragments by sonication (200–600 base pairs) and sequenced on Illumina HiSeq-2500 platform (PE 125 bp) with the paired-end module. About 110.96 Gb of raw data were obtained from *L. dispar asiatica* (Table 3).

**Table 3:** Statistics of the Hic sequence data used for genome assembly.

Sample	Raw paired reads	Raw Base(bp)	Duplication rate(%)	Effective Rate(%)	Error Rate(%)	Q20(%)	Q30(%)	GC Content(%)
*L. dispar asiatica* larvae	36,98,62,582	1,10,95,87,74,600	20.31%	78.97	0.03	97.47	93.23	40.02

**Fig. 1:**
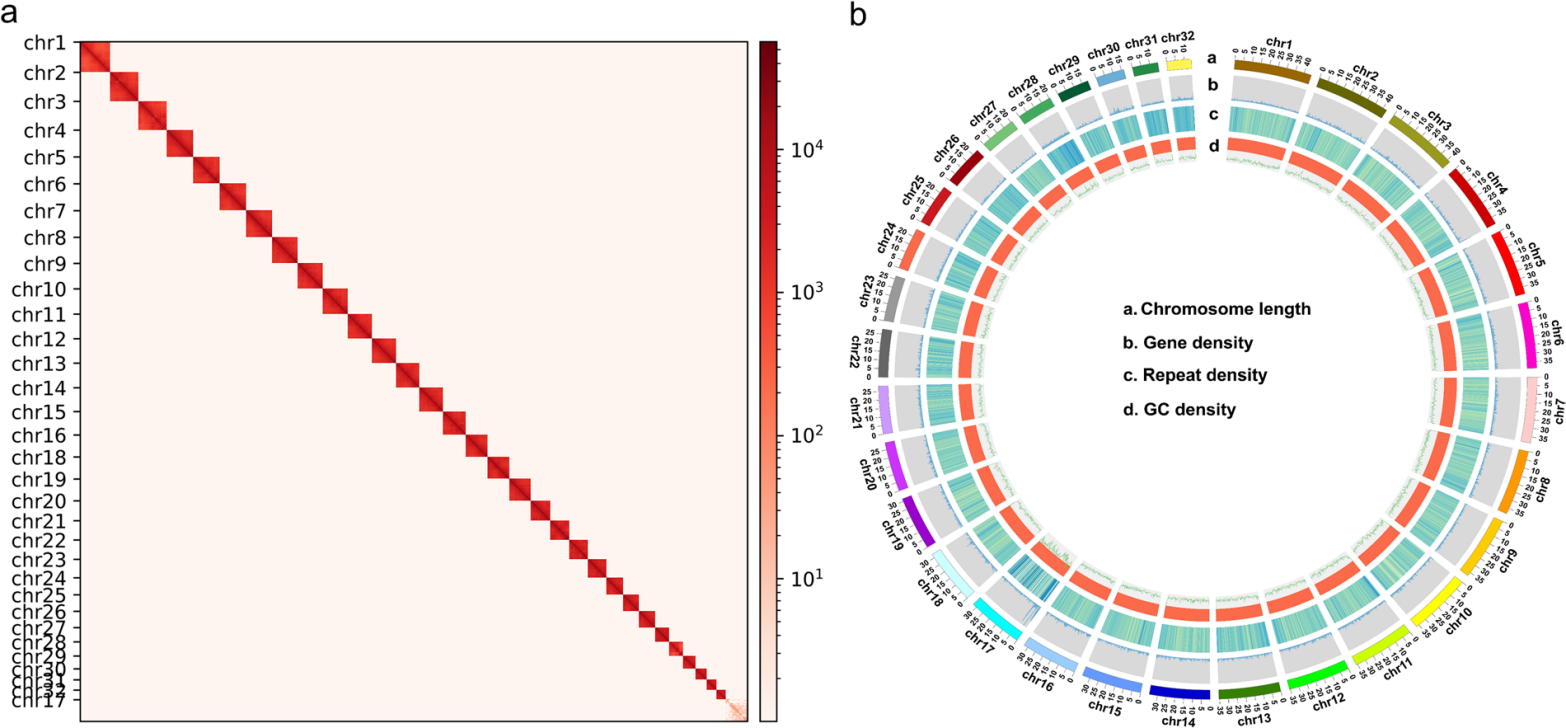
The genome features of *Lymantria dispar asiatica*. (**a**) genome-wide Hi-C heatmap of chromatin interaction counts. (**b**) Circos plot of the 32 chromosomes of *Lymantria dispar asiatica*. From the outermost layer to the innermost layer, the chromosome length, gene density, repeat density, and GC density are sequentially displayed.

The high-quality sequencing reads were filtered by fastp v0.23.4^20^. The cleaned Hi-C reads were then mapped to the draft genome using Juicer v1.6^21^. The unique high-quality paired-end reads were taken as input for 3D-DNA v190716 pipeline^22^ with parameters “-r 0”. Chromosome interaction matrix was manually adjusted by using JuicerBox v1.11.08^21^. The Hi-C heatmap was drawn with HiCExplorer v3.7.2^23^. Finally, a total of 32 chromosomes was obtained, which contained 99.38% of the assembled contigs (Fig. 1).

After Hi-C scaffolding, the genome integrity was evaluated using Benchmarking Universal Single-Copy Orthologs (BUSCO v5.4.3)^24^. This analysis revealed that *L. dispar asiatica* chromosome level assembly contained C:99.4% [S:97.9%, D:1.5%], F:0.2%, M:0.4%, n:1367 (Table 4). The results indicated that the genome assembly of *L. dispar asiatica* were of considerable contiguity, completeness and accuracy.

**Table 4:** Statistics of repetitive elements in the Lymantria disapr asiatica genome.

Elements	Repeat type	Number of elements	length (bp)	percentage in genome
SINEs	Retroelements	331135	22932667	2.30%
Penelope	Retroelements	0	0	0.00%
LINEs	Retroelements	1188736	242743224	24.33%
CRE/SLACS	Retroelements	640	105470	0.01%
L2/CR1/Rex	Retroelements	98136	21226467	2.13%
R1/LOA/Jockey	Retroelements	138973	73208592	7.34%
R2/R4/NeSL	Retroelements	14042	2586546	0.26%
RTE/Bov-B	Retroelements	632322	90599808	9.08%
L1/CIN4	Retroelements	227	17956	0.00%
BEL/Pao	Retroelements	11923	8500706	0.85%
Ty1/Copia	Retroelements	7017	1982373	0.20%
Gypsy/DIRS1	Retroelements	28427	10317472	1.03%
Retroviral	Retroelements	3967	281397	0.03%
hobo-Activator	DNA transposons	140277	18453863	1.85%
Tc1-IS630-Pogo	DNA transposons	91034	26313765	2.64%
En-Spm	DNA transposons	0	0	0.00%
MULE-MuDR	DNA transposons	994	78938	0.01%
PiggyBac	DNA transposons	7452	1244764	0.12%
Tourist/Harbinger	DNA transposons	5026	808317	0.08%
Other (Mirage, P-element, Transib)	DNA transposons	2115	256393	0.03%
Rolling-circles	Rolling-circles	564710	78046078	7.82%
Satellites	Small RNA	5385	524461	0.05%
Simple repeats	Small RNA	188889	9526362	0.95%
Low complexity	Small RNA	23215	1097031	0.11%
Unclassified:		792468	88703522	8.89%

### RNA sequencing

Larvae in the 2nd, 3rd, 4th, and 5th instars, along with adult females, were collected for RNA extraction. Total RNA was extracted from each tissue respectively using TRIzol reagent. Sequencing libraries were generated by NEBNext Ultra RNA Library Prep Kit (NEB, USA). The transcriptomes were sequenced on the Illumina Hiseq 4000 platform with PE150 strategy, and a total of 33.99 Gb short-read RNA-seq raw data were obtained (Table 5).

**Table 5:** Statistics of RNA sequcing data of Lymantria disapr asiatica.

Sample	Raw Reads	Clean Reads	Raw Base(G)	Clean Base(G)	Effective(%)	Error(%)	Q20(%)	Q30(%)	GC(%)
2 instar	49084562	43903568	7.36	6.59	89.44	0.03	97.74	93.6	45.02
3 instar	50192094	46752784	7.53	7.01	93.15	0.03	97.6	93.27	43.41
4 instar	44017684	41351054	6.6	6.2	93.94	0.03	97.46	92.96	43.37
5 instar	44119946	40964012	6.62	6.14	92.85	0.03	97.79	93.73	42.71
Adult female	39174758	36613146	5.88	5.49	93.46	0.03	97.67	93.55	45.17

### Genome annotation

For insect genome annotation, we mainly referred to a genome annotation pipeline developed by the Institute of Insect Sciences, Zhejiang University^25^. In detail, a de novo repeat library of insect specialization was firstly constructed using RepeatModeler v2.0.4^26^ and RepeatMasker v4.1.5^27^ for repeat sequence annotation. Then the genome was masked by RepeatMasker v4.1.5 with “-xsmall” parameters. In the genomic sequences, a total of 527.67 Mb (52.89%) repetitive elements were identified, mainly including 28.98% retroelements, 5.94% DNA transposons, 7.82% rolling-circles, and 8.89% unclassified repeat sequence (Table 2). To predict protein-coding genes, homology proteins were obtained from other insect species (downloaded from InsectBase 2.0^25^). Transcriptome data was aligned to the genome using HISAT v2.2.1^28^ and the open reading frame (ORF) was predicted using StringTie v2.2.1^29^ combined with TransDecoder v5.7.0^30^. Both homology proteins and transcriptome-based evidence were as inputs to BRAKER v3.0.3^31^, which containing *ab initio* gene prediction generated by Augustus v3.4.0^32^ and Genemark-ETP mode v1.0^33^. A total of 19,532 protein-coding genes was predicted in the genome of *L. dispar asiatica*. Functional annotation of protein-coding genes was evaluated based on eggNOG-mapper v2 (http://eggnog-mapper.embl.de/)^34^.

## Data Records

Illumina, PacBio and Hi-C raw data for *L. dispar asiatica* genome sequencing have been deposited in the NCBI Sequence Read Archive with accession number SRR26057469^35^, SRR26036511^36^ and SRR2604630^37^. Illumina transcriptome data for larvae and adult have been deposited in the NCBI Sequence Read Archive with accession number SRP459597^38^. The final assembled *L. dispar asiatica* genome has been submitted to the GenBank database of NCBI with accession number GCA_032191425.1^39^. The annotation file is available in figshare^40^.

## Technical Validation

The completeness of *L. dispar asiatica* genome assembly was evaluated using the BUSCO (in the insects_odb10 database), and the completeness was 99.40% (97.9% single-copied genes and 1.5% duplicated genes), 0.2% fragmented, and 0.4% missing genes. The Hi-C heatmap revealed a well-structured interaction pattern in and around the chromosome inversion regions, with the notable exception of chromosome 17. This chromosome showed a lower probability of contact compared to others, leading to speculation that it may be associated with the W sex chromosome, which is specific to females. Besides, the mapping rates of short-reads sequencing data exceeds 90%. All evidence strongly supported that the completeness and accuracy of *L. dispar asiatica* genome assembly.

## Data Availability

This study did not employ a custom script; data processing was conducted following the protocols and manuals of the relevant bioinformatics software mentioned in Methods section.
